# Evolutionary Analysis and Expression Profiling of the TIFY Gene Family in Banana Under Multiple Stresses and Functional Characterization of *MaTIFY20* in Drought Tolerance

**DOI:** 10.3390/ijms27157044

**Published:** 2026-08-06

**Authors:** Sheraz Ahmad, Huimin Song, Hangbo Cao, Jingjing Liu, Rahat Sharif

**Affiliations:** 1 Guangdong Provincial Key Lab of Agro-Animal Genomics and Molecular Breeding, College of Animal Science, South China Agricultural University, Guangzhou 510642, China; dh20010@yzu.edu.cn; 2 Department of Pomology, College of Horticulture, South China Agricultural University, Guangzhou 510642, China

**Keywords:** *Musa acuminata*, TIFY, jasmonic acid, drought stress, gene expression, *MaTIFY20*

## Abstract

The TIFY gene family comprises plant-specific transcriptional regulators central to jasmonic acid (JA) signaling and responses to biotic and abiotic stresses. Despite the economic importance of the banana (*Musa* spp.), the TIFY family remains largely uncharacterized in this crop. Here, we conducted a genome-wide identification and comprehensive analysis of the *MaTIFY* gene family in *Musa acuminata*. A total of 47 *MaTIFY* genes were identified, distributed across all 11 chromosomes. Phylogenetic analysis classified these into four subfamilies (TIFY, ZIZ/ZML, PPD, and JAZ), and conserved motif and domain analyses revealed a core TIFY domain architecture with subfamily-specific structural features. Gene Ontology (GO) enrichment and *cis*-acting regulatory element analyses suggested potential involvement in JA-mediated signaling, defense response, and hormone cross-talk. Expression profiling under drought, *Fusarium oxysporum* f. sp. *cubense* race 4 (*Foc* 4), and cold stress revealed distinct transcriptional responses, with *MaTIFY5*, *MaTIFY16*, *MaTIFY20*, *MaTIFY26*, and *MaTIFY30* exhibiting enhanced induction in resistant cultivars compared to their susceptible counterparts. Functional characterization of *MaTIFY20* confirmed its significant upregulation under drought stress and its ability to confer enhanced osmotic tolerance when heterologously expressed in yeast. These findings provide novel insights into the evolutionary dynamics and stress-responsive functions of banana TIFY genes and identify candidate targets for molecular breeding to improve abiotic and biotic stress resilience in banana.

## 1. Introduction

As lipid-derived phytohormones, jasmonic acid (JA) and its biologically active derivatives share structural similarities with prostaglandins. Initially recognized primarily as a plant growth inhibitor [1], JA was later found to play a crucial role in orchestrating plant defenses against both biotic challenges, such as pathogen infections and insect herbivory, and abiotic stresses, including drought, salinity, and temperature extremes [2,3]. Furthermore, it governs a wide array of developmental processes, encompassing fertility, root architecture, leaf senescence, flowering, fruit maturation, and the biosynthesis of secondary metabolites [4,5].

Central to JA signaling is the COI1-dependent degradation of TIFY repressors [6,7]. Upon binding bioactive jasmonoyl-L-isoleucine (JA-Ile), COI1, an E3 ubiquitin ligase component, targets these repressors for proteasomal removal, thereby freeing MYB and MYC transcription factors to drive downstream gene expression. In contrast, in the absence of JA, TIFY proteins keep these transcriptional activators tightly repressed through direct physical interactions [8,9]. Notably, TIFY family members exhibit diverse functions beyond transcriptional repression; they assemble into homo- and heteromeric complexes with family members and transcription factors, thereby fine-tuning JA-responsive outputs and facilitating hormonal crosstalk [7,10]. Consequently, probing the inner workings of these conserved hubs is fundamental to unveiling the functional breadth, specificity, and genetic redundancy of the TIFY family, as well as the complex regulatory networks that mediate the plant’s priority-setting between growth and immunity.

Researchers have used genome-wide analyses to identify TIFY gene families across numerous plant species, uncovering substantial variation in family size. These families contain 18 genes in *Arabidopsis thaliana* [11], 20 in *Oryza sativa* [12], 30 in *Zea mays*, 16 in *Capsicum annuum*, 21 in *Juglans regia*, 16 in *Camellia sinensis*, and 25 in *Populus trichocarpa* [13].

Banana (*Musa* spp.) is a large herbaceous monocot and the fourth most essential food crop in developing countries, according to the FAO [14]. Its economic significance is particularly evident in China, the world’s second-largest producer, which yielded 11.77 million tons across 505,000 hectares in 2022 [15]. Despite these impressive production figures, the banana industry faces substantial agronomic challenges. Due to their shallow root systems, banana plants require consistent and abundant water [16,17]. While major cultivation regions, including Taiwan, Guangxi, and Fujian, benefit from high annual rainfall, they are still prone to seasonal water deficits during the spring and autumn. This vulnerability is further magnified by global climate change, which has increased the frequency of irregular droughts and made water scarcity a primary limiting factor for the sustainable growth of China’s banana production. TIFY genes have been previously characterized for their role in drought stress tolerance [18,19].

Therefore, in this study, we conducted a genome-wide identification and characterization of the banana TIFY gene family, including an analysis of phylogeny, conserved motifs, and *cis*-regulatory elements. Additionally, we examined the expression of *MaTIFY* genes under drought, cold, and *Foc* 4 stress. Additionally, we performed heterologous overexpression of *MaTIFY20* and examined its role in drought tolerance. Ultimately, this work provides a theoretical basis and identifies candidate genes to support molecular breeding for enhanced drought stress tolerance in banana.

## 2. Results

### 2.1. Identification of the TIFY Gene Family in Banana

The 47 *MaTIFY* genes were distributed across all 11 chromosomes (Table 1). The family showed considerable size variation (Figure S1a), with MaTIFY2 being the largest protein (436 aa, 46.6 kDa). MaTIFY38 is the smallest (105 aa, 10.8 kDa), and nearly half (48.9%) of the proteins fell within the medium size range (20–40 kDa). A strong bias toward basic proteins was observed, as 78.7% had a pI > 7.5 with only nine acidic members (pI < 7.0); the most basic protein was MaTIFY15 (pI = 10.70) and the most acidic was MaTIFY17 (pI = 5.91). All 47 proteins were predicted to localize to the nucleus (Figure S1b), which is consistent with the TIFY family functioning as transcription factors involved in jasmonate signaling and stress responses. CDS length and protein length showed a perfect correlation (r = 1.000), and CDS length strongly correlated with molecular weight (r = 0.998) (Figure S1c). Notably, there was a weak correlation between protein size and pI (r ≈ −0.08), indicating that these properties evolved independently. Furthermore, multiple clusters of closely spaced genes were identified on chromosomes 3, 5, 6, 8, and 9, supporting duplication-driven expansion of this gene family.

### 2.2. Chromosomal Distribution

**Figure 1:**
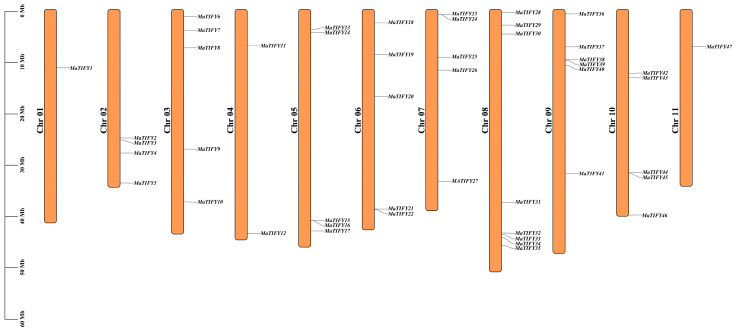
Chromosomal distribution of *MaTIFY* genes on the 11 banana (*M. acuminata*) chromosomes.

The chromosomal localization analysis revealed that the 47 identified *MaTIFY* genes are distributed across all 11 chromosomes of banana (Figure S1d), with an uneven distribution pattern (Figure 1). The number of *MaTIFY* genes per chromosome varied considerably. Chromosome 8 harbored the highest number with eight genes (*MaTIFY28*, *MaTIFY29*, *MaTIFY30*, *MaTIFY31*, *MaTIFY32*, *MaTIFY33*, *MaTIFY34*, *MaTIFY35*), followed by Chromosome 9 with 6 genes (*MaTIFY36*, *MaTIFY37*, *MaTIFY38*, *MaTIFY39*, *MaTIFY40*, *MaTIFY41*). Chromosomes 3, 5, 6, 7, and 10 each contained five genes (*MaTIFY6–MaTIFY10*; *MaTIFY13–MaTIFY17*; *MaTIFY18–MaTIFY22*; *MaTIFY23–MaTIFY27*; and *MaTIFY42–MaTIFY46*, respectively). Chromosome 2 carried 3 genes (*MaTIFY3*, *MaTIFY4*, *MaTIFY5*), while Chromosomes 1 and 4 each contained 2 genes (*MaTIFY1*, *MaTIFY2* and *MaTIFY11*, *MaTIFY12*, respectively). Chromosome 11 had only 1 gene (*MaTIFY47*). Notably, several gene clusters were observed, including a cluster of four genes (*MaTIFY32–MaTIFY35*) at approximately 45 Mb on Chromosome 8, a cluster of two genes (*MaTIFY23–MaTIFY24*) at the top of Chromosome 7, and multiple tandem arrays such as *MaTIFY13/MaTIFY14* on Chromosome 5 and *MaTIFY42/MaTIFY43* on Chromosome 10. The physical positions of these genes spanned the entire length of each chromosome, with no apparent preference for centromeric or telomeric regions. This uneven chromosomal distribution and the presence of gene clusters suggest that tandem and segmental duplications may have contributed to the expansion of the TIFY gene family during banana genome evolution.

### 2.3. Evolutionary Analysis

**Figure 2:**
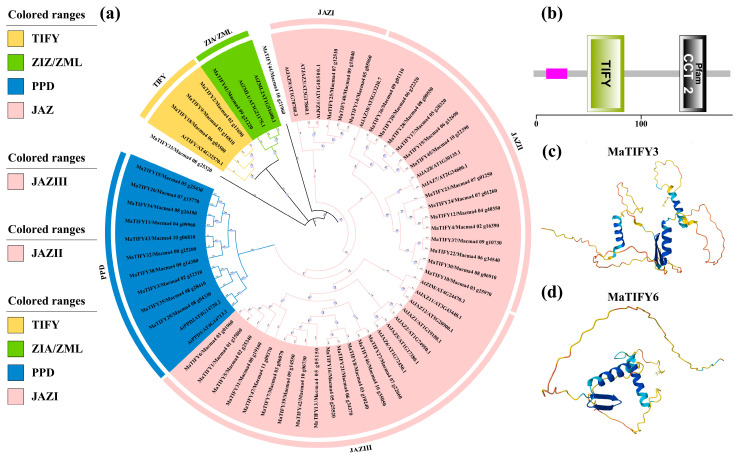
Phylogenetic analysis, domain architecture, and three-dimensional structure prediction of MaTIFY proteins. (**a**) Neighbor-joining phylogenetic tree of TIFY proteins from *M. acuminata* (MaTIFY) and *Arabidopsis thaliana* (At). The tree is divided into four subfamilies: TIFY (yellow), ZIZ/ZML (green), PPD (blue), and JAZ (pink). The JAZ subfamily is further subdivided into three groups: JAZI, JAZII, and JAZIII. Bootstrap values are indicated at the nodes. (**b**) Schematic representation of the conserved domain architecture showing the N-terminal TIFY domain (PF06200, green) and the C-terminal CCT_2 domain (PF06203, gray) characteristic of specific subfamily members. (**c**,**d**) Predicted three-dimensional homology models of representative MaTIFY3 (**c**) and MaTIFY6 (**d**) proteins, displaying the conserved α/β-fold architecture with α-helices (blue), β-sheets (yellow), and coil/loop regions (orange). The TIFY domain forms a compact globular structure in both proteins.

To elucidate the evolutionary relationships and subfamily classification of banana TIFY proteins, a neighbor-joining phylogenetic tree was constructed using the full-length amino acid sequences of *M. acuminata* TIFY (MaTIFY) proteins alongside their Arabidopsis thaliana orthologs. The analysis partitioned the TIFY superfamily into four distinct subfamilies: TIFY (yellow), ZIZ/ZML (green), PPD (blue), and JAZ (pink), consistent with the established classification in *A. thaliana*. The JAZ subfamily represented the largest clade and was further divided into three subgroups: JAZI, JAZII, and JAZIII (Figure 2a). While JAZI and JAZII contained the majority of members, JAZIII was relatively small. Notably, most MaTIFY proteins clustered closely with their *A. thaliana* counterparts, indicating high sequence conservation and probable functional orthology. For example, MaTIFY3 and MaTIFY6 were grouped within the TIFY subfamily with AtTIFY1, while various MaTIFY genes were distributed across the three JAZ subclades. Domain architecture analysis revealed that all members contain the canonical N-terminal TIFY domain (PF06200) (Figure 2b). Additionally, a CCT_2 domain (PF06203) was present in specific subfamily members, suggesting functional diversification through domain shuffling. To explore their structural features, three-dimensional (3D) homology models were generated for representative proteins. The predicted structures of MaTIFY3 (Figure 2c) and MaTIFY6 (Figure 2d) both displayed an α/β α/β-fold architecture characterized by multiple α-helices (blue) and coil/loop regions (yellow/orange), with the TIFY domain forming a compact globular structure. The high structural similarity between these two proteins reflects a conserved structural scaffold within the TIFY subfamily. Collectively, these results validate the identification and phylogenetic classification of the MaTIFY gene family and provide a structural foundation for understanding their functional mechanisms in banana.

### 2.4. Conserved Motif Composition of MaTIFY Proteins

**Figure 3:**
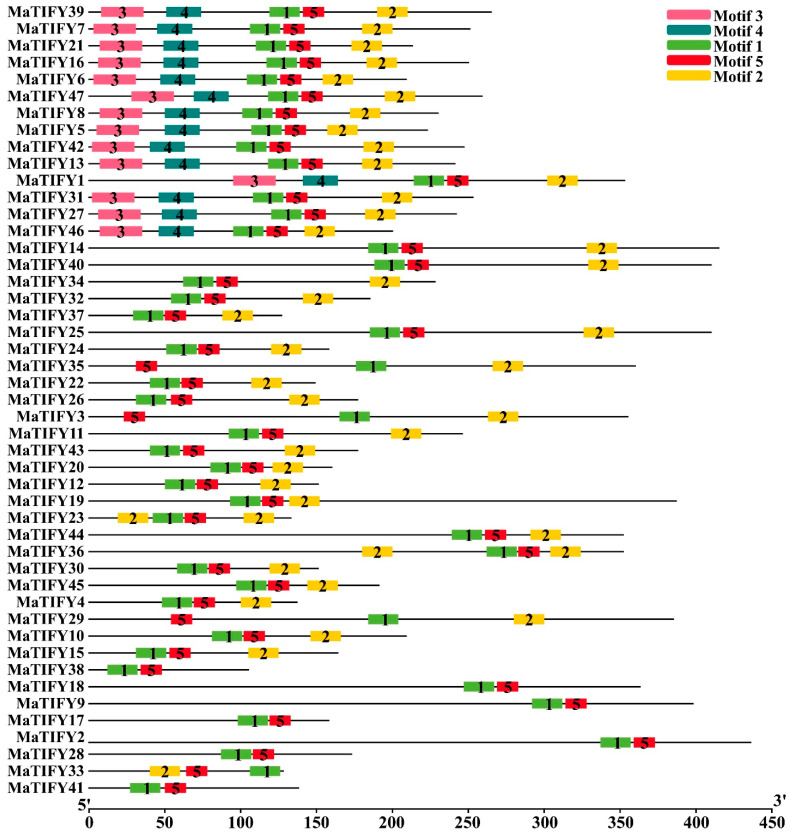
Conserved motif composition of MaTIFY proteins. The distribution of five conserved motifs (Motif 1–Motif 5), identified by MEME analysis, is mapped across the full-length protein sequences of 47 MaTIFY members.

To investigate the structural diversity and conserved sequence features within the banana TIFY family, MEME motif analysis was performed on the full-length protein sequences of all 47 MaTIFY members. A total of five conserved motifs, designated Motif 1 through Motif 5, were identified and mapped across the protein architectures (Figure 3). Motif 1 (green) was the most widely distributed element, present in 43 of the 47 proteins, and occupied a central position within the primary sequence. Motif 5 (red) was also highly prevalent and frequently localized immediately adjacent to Motif 1, suggesting that these two motifs may constitute a functionally coupled module. Motif 2 (yellow) was detected in the majority of members, typically situated toward the C-terminal region. In contrast, Motif 3 (pink) and Motif 4 (teal) were restricted to a subset of proteins and were predominantly positioned at the N-terminal end. Based on the arrangement of these motifs, the MaTIFY proteins could be broadly classified into several architectural groups. The first group, comprising MaTIFY39, MaTIFY7, MaTIFY21, MaTIFY16, MaTIFY6, MaTIFY8, MaTIFY5, MaTIFY42, MaTIFY13, MaTIFY1, MaTIFY31, MaTIFY27, and MaTIFY46, exhibited the completely conserved architecture of Motif 3–Motif 4–Motif 5–Motif 2 in the N- to C-terminal direction. A second group, including MaTIFY14, MaTIFY40, MaTIFY34, MaTIFY32, MaTIFY37, MaTIFY25, MaTIFY24, MaTIFY35, MaTIFY22, MaTIFY26, MaTIFY3, MaTIFY11, MaTIFY43, MaTIFY20, MaTIFY12, MaTIFY19, and MaTIFY23, displayed variations in this architecture, often lacking Motif 3 and/or Motif 4 while retaining the Motif 1–Motif 5 core. Notably, MaTIFY1 possessed a distinctive bipartite structure containing two separate Motif 1–Motif 5 modules. A third group, represented by MaTIFY38, MaTIFY9, MaTIFY17, MaTIFY2, MaTIFY28, MaTIFY33, and MaTIFY41, exhibited a highly simplified motif composition, harboring only Motif 1 and Motif 5 (or minimal variants thereof), which is consistent with the domain architecture characteristic of the TIFY and ZIZ/ZML subfamilies. Additionally, several proteins, such as MaTIFY36 and MaTIFY44, contained duplicated instances of Motif 2 (Figure 3).

### 2.5. Gene Ontology Enrichment Analysis of MaTIFY Genes

**Figure 4:**
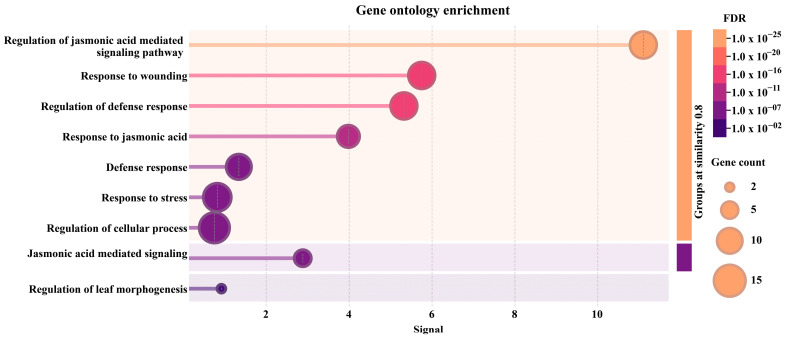
Gene Ontology (GO) enrichment analysis of *MaTIFY* genes. Significantly enriched biological process terms are displayed as a dot plot, with the x-axis representing enrichment signal intensity and the y-axis indicating the GO term. Circle size corresponds to the number of annotated genes, and color intensity reflects the false discovery rate (FDR) value.

To gain insight into the putative biological functions of the banana TIFY gene family, Gene Ontology (GO) enrichment analysis was performed on all 47 MaTIFY members. The significantly enriched biological process terms were predominantly associated with hormone signaling, stress response, and defense regulation (Figure 4). The most significantly enriched term was regulation of jasmonic-acid-mediated signaling pathway, which exhibited the highest enrichment signal (approximately 11.0), the largest gene count (15 genes), and the strongest statistical significance (FDR ≈ 1.0 × 10^−25^). This was followed by a response to wounding and regulation of defense response, both of which displayed substantial enrichment signals (~6.0 and ~5.5, respectively), involved approximately 10 genes each, and reached highly significant FDR values (≈1.0 × 10^−16^). “Response to jasmonic acid” also represented a major enriched category, with a signal of ~4.0, 5 annotated genes, and an FDR of ≈1.0 × 10^−11^. Additional enriched terms included defense response, response to stress, and regulation of cellular process, which showed comparatively lower enrichment signals (~1.2–1.5) but remained statistically significant (FDR ≈ 1.0 × 10^−7^). Jasmonic acid-mediated signaling and regulation of leaf morphogenesis were detected with smaller gene sets (2 genes each) and moderate enrichment signals (~3.0 and ~0.8, respectively). Semantic similarity clustering (similarity cutoff = 0.8) grouped these GO terms into two primary functional categories. The first cluster (highlighted in orange) comprised the top four terms centered on jasmonic acid signaling regulation, wounding response, and defense response regulation, indicating a tightly interconnected functional module. The second cluster (highlighted in purple) encompassed basal defense response, general stress response, cellular process regulation, and leaf morphogenesis, representing broader physiological processes. Collectively, these enrichment patterns suggest that the MaTIFY gene family in banana may be involved in jasmonate-mediated signaling networks and plant defense responses, consistent with the established functions of TIFY family genes in other plant species.

### 2.6. Protein–Protein Interaction Network Analysis of MaTIFY Proteins

**Figure 5:**
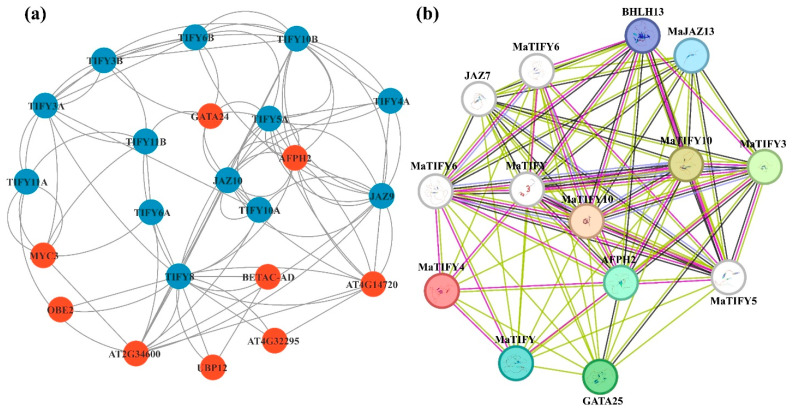
Protein–protein interaction (PPI) network analysis of TIFY proteins. (**a**) Arabidopsis thaliana TIFY interaction network showing TIFY family members (blue nodes) and non-family interaction partners (red nodes). (**b**) Predicted banana TIFY PPI network displaying a topology highly reminiscent of the Arabidopsis network. Central banana TIFY nodes connect with orthologous partners.

To explore the functional association landscape of banana TIFY proteins, protein–protein interaction (PPI) network analysis was performed using the Arabidopsis TIFY interaction map as a reference. In the Arabidopsis network (Figure 5a), TIFY family members (blue nodes) formed a densely interconnected web with both paralogous TIFY proteins and non-family interaction partners (red nodes). TIFY8 emerged as a prominent hub, establishing extensive connections with multiple TIFY paralogs as well as with GATA24, AFP2, and UBP12. JAZ9 and JAZ10 also occupied central positions, linking to transcriptional regulators such as MYC3, OBE2, and AT2G34600. Additional interactors included the developmental proteins BETAC-AD and AT4G32295, and the ubiquitin-associated protein AT4G14720, suggesting that Arabidopsis TIFY proteins function within transcriptional and ubiquitin-mediated regulatory modules.

The predicted banana TIFY PPI network (Figure 5b) displayed a topology highly similar to the Arabidopsis network, indicating substantial conservation of interaction partnerships across the two species. Key banana TIFY proteins, including MaTIFY6, MaTIFY10, MaJAZ13 (a TIFY gene belonging to JAZ subclades), MaTIFY3, and MaTIFY5, were positioned as central nodes, establishing connections with orthologous partners such as BHLH13 (a bHLH transcription factor likely corresponding to the MYC3 ortholog), GATA25 (a GATA transcription factor), AFP2, and JAZ7. MaTIFY4 and GATA25 appeared as relatively peripheral nodes with fewer connections. The dense interconnections among MaTIFY proteins, together with their predicted associations with transcription factors and signaling components, support the hypothesis that banana TIFY genes operate within conserved jasmonate signaling and transcriptional regulatory cascades.

In addition to protein–protein interactions, certain MaTIFY genes are co-regulated by multiple miRNAs. Specifically, MaTIFY21 is targeted by miR164a and miR171a, while miR171a and miR408 target MaTIFY20. MaTIFY13 and MaTIFY26 appear to be targeted by specific miRNAs (miR408 and miR164a, respectively), indicating specialized regulatory roles (Figure S2).

### 2.7. Cis-Acting Regulatory Element and Expression Profile Analysis of MaTIFY Genes

**Figure 6:**
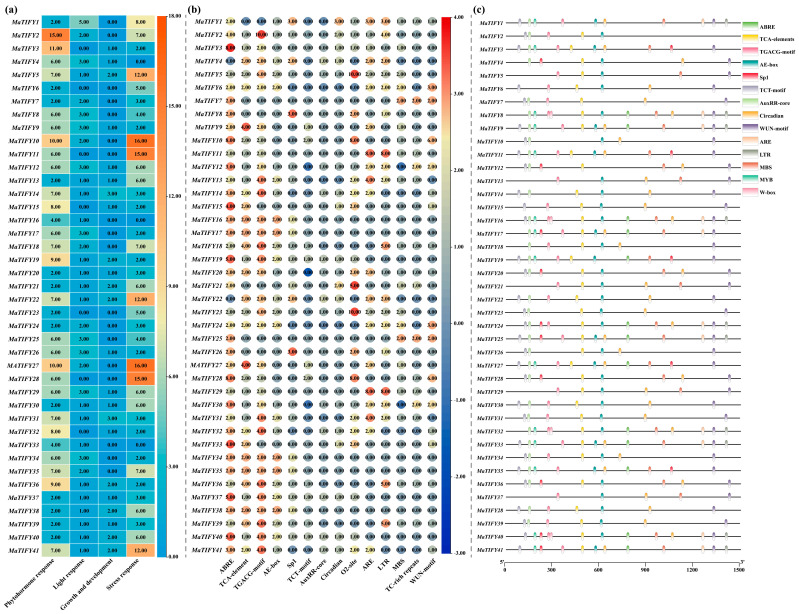
*Cis*-acting regulatory element analysis of MaTIFY gene promoters. (**a**) Heatmap showing the cumulative counts of *cis*-elements categorized into three functional classes: phytohormone response, light response, and growth and development/stress response across 41 MaTIFY promoters. Color intensity indicates the total element count per gene. (**b**) Detailed heatmap displaying the abundance of individual *cis*-elements. Circle size and color intensity reflect element abundance. (**c**) Schematic distribution of specific *cis*-elements along the 1500-bp promoter region (5′ to 3′) of each *MaTIFY* gene.

To investigate the potential regulatory mechanisms governing MaTIFY gene expression, the 1500-bp promoter regions upstream of the transcription start sites were analyzed for the presence of cis-acting regulatory elements. These classes included phytohormone response, light response, and growth and development/stress response (Figure 6a,b). Phytohormone-responsive elements were highly abundant across the MaTIFY promoters, with abscisic acid-responsive elements (ABRE) being the most prevalent, followed by methyl jasmonate-responsive elements (TGACG-motif and CGTCA-motif), salicylic acid-responsive elements (TCA-elements), and auxin-responsive elements (AuxRR-core and TGA-element). Notably, *MaTIFY12* and *MaTIFY18* harbored the highest numbers of phytohormone-responsive elements (15.00 and 16.00, respectively), whereas *MaTIFY4*, *MaTIFY15*, and *MaTIFY28* contained markedly fewer (0.00–3.00). Light-responsive elements, including G-box, GT1-motif, and TCT-motif, were ubiquitously present but showed relatively uniform distribution among family members. Growth and development/stress-responsive elements, such as those associated with drought (MBS), low temperature (LTR), and wound response (WUN-motif), were also identified, with *MaTIFY12*, *MaTIFY18*, and *MaTIFY41* exhibiting the highest cumulative scores (7.80–12.00). The distribution of specific *cis*-elements across individual promoters revealed distinct regulatory signatures (Figure 6c). ABRE and MBS elements were widely distributed, suggesting broad involvement of *MaTIFY* genes in ABA-mediated and drought stress signaling pathways. MYB-binding sites (MRE and MYB) and W-box elements were also frequently detected, implying transcriptional regulation by MYB and WRKY transcription factors. Circadian elements and ARE (anaerobic response elements) were present in a subset of promoters, indicating potential roles in circadian rhythm regulation and hypoxia response. The differential composition and abundance of these *cis*-elements among *MaTIFY* members suggest that individual genes are subject to distinct regulatory inputs, which may underlie their functional specialization in response to diverse environmental and hormonal cues.

### 2.8. Expression Profiles of MaTIFY Genes Under Drought, Foc 4, and Cold Stress Conditions

**Figure 7:**
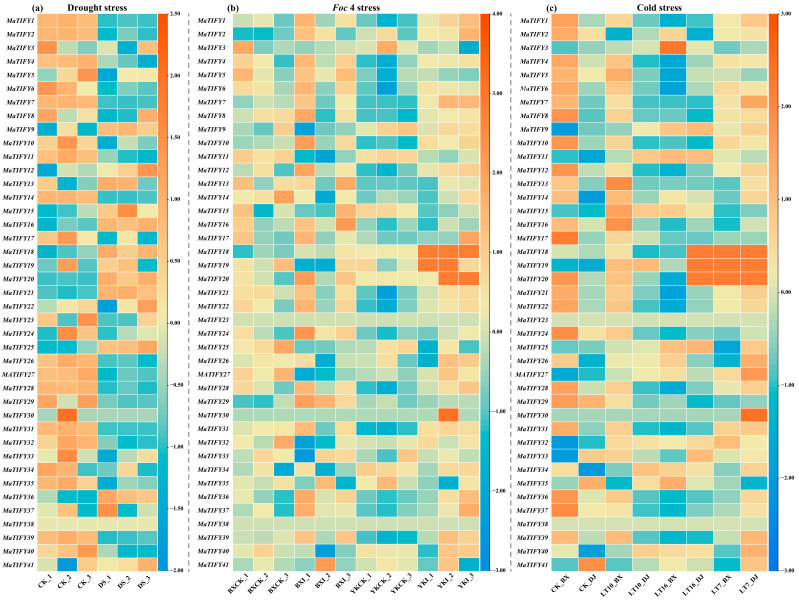
Expression profiles of *MaTIFY* genes under drought, *Foc 4*, and cold stress conditions. Heatmaps display the normalized expression levels of 41 MaTIFY genes under (**a**) drought stress (CK: control; DS: drought stress), (**b**) *Foc* 4 infection in susceptible Baxijiao (BX) and resistant Yueyoukang1 (YK) cultivars (CK: control; I: infected), and (**c**) low-temperature treatment in cold-sensitive Baxijiao (BX) and cold-tolerant Dajiao (DJ) cultivars at 16 °C (LT16), 10 °C (LT10), and 7 °C (LT7). The color scale indicates relative expression levels, with red/orange indicating upregulation and blue indicating downregulation. The remaining six MaTIFY genes showed no detectable expression in the dataset.

To investigate the transcriptional responses of banana TIFY genes to abiotic and biotic stresses, the expression patterns of 41 *MaTIFY* genes were analyzed under drought (Figure 7a), *Foc* 4 (Figure 7b), and cold stress conditions (Figure 7c) using transcriptomic data. The remaining six genes showed no expression in our dataset. Under drought stress (Figure 7a), the *MaTIFY* genes exhibited diverse expression patterns. A subset of genes, including *MaTIFY3*, *MaTIFY5*, *MaTIFY9*, *MaTIFY16*, *MaTIFY20*, and *MaTIFY25*, displayed upregulation in drought-treated samples (DS) compared to controls (CK). Conversely, *MaTIFY11*, *MaTIFY14*, *MaTIFY23*, *MaTIFY27*, *MaTIFY32*, *MaTIFY33*, *MaTIFY34*, and *MaTIFY37* were significantly downregulated under drought conditions. Several genes, such as *MaTIFY8*, *MaTIFY13*, *MaTIFY17*, *MaTIFY22*, *MaTIFY24*, *MaTIFY35*, *MaTIFY36*, *MaTIFY38*, and *MaTIFY41* showed relatively stable or modestly altered expression levels.

Under *Foc* 4 infection (Figure 7b), the expression patterns of MaTIFY genes were compared between the susceptible Baxijiao (BX) and the highly resistant Yueyoukang1 (YK) banana cultivars. Distinct genotype-specific transcriptional responses were evident upon pathogen challenge. In the susceptible BX cultivar, several genes, including *MaTIFY5*, *MaTIFY6*, *MaTIFY10*, *MaTIFY16*, *MaTIFY26*, and *MaTIFY29*, exhibited marked upregulation in infected samples (BXI) relative to controls (BXCK). Conversely, *MaTIFY3*, *MaTIFY9*, *MaTIFY12*, *MaTIFY19*, *MaTIFY21*, *MaTIFY23*, *MaTIFY32*, *MaTIFY33*, and *MaTIFY34* were significantly downregulated in BX upon *Foc* 4 infection. In the resistant YK cultivar, a distinct expression signature was observed. Genes such as *MaTIFY5*, *MaTIFY6*, *MaTIFY10*, *MaTIFY16*, *MaTIFY20*, *MaTIFY26*, *MaTIFY29*, and *MaTIFY30* showed strong and sustained upregulation in infected samples (YKI) compared to controls (YKCK), potentially contributing to the robust defense phenotype. Notably, the magnitude and consistency of upregulation of these genes were more pronounced in YK than in BX, suggesting that differential activation of these MaTIFY genes may underlie the resistance mechanism. Additionally, *MaTIFY3*, *MaTIFY9*, *MaTIFY12*, *MaTIFY23*, *MaTIFY32*, *MaTIFY33*, and *MaTIFY34* were downregulated in YK following *Foc* 4 challenge, though the extent of repression differed between the two cultivars. The comparative analysis between BX and YK revealed both conserved and divergent *MaTIFY* expression patterns. The shared upregulation of *MaTIFY5*, *MaTIFY6*, *MaTIFY10*, *MaTIFY16*, and *MaTIFY29* in both genotypes suggests their fundamental roles in basal defense activation. However, the enhanced and prolonged induction of these genes in the resistant YK cultivar, coupled with the differential repression of negative regulators, indicates that the amplitude and dynamics of MaTIFY-mediated transcriptional reprogramming are critical determinants of disease resistance. These findings implicate specific *MaTIFY* genes as potential contributors to the differential *Foc* 4 resistance between BX and YK bananas and provide candidate targets for breeding programs aimed at enhancing banana resistance to Fusarium wilt.

Under low-temperature treatment (Figure 7c), the transcriptional responses of MaTIFY genes were evaluated in the cold-sensitive Baxijiao (BX) and the cold-tolerant Dajiao (DJ) banana across a temperature gradient of 16 °C (LT16), 10 °C (LT10), and 7 °C (LT7). Marked genotype-specific differences in cold-responsive expression were observed between the two cultivars. In the cold-sensitive BX cultivar, progressive downregulation was detected for *MaTIFY3*, *MaTIFY9*, *MaTIFY12*, *MaTIFY14*, *MaTIFY18*, *MaTIFY19*, *MaTIFY23*, *MaTIFY27*, *MaTIFY32*, *MaTIFY33*, *MaTIFY34*, and *MaTIFY40*, as temperatures decreased, with the strongest repression generally occurring at 7 °C (LT7_BX). Conversely, *MaTIFY5*, *MaTIFY6*, *MaTIFY7*, *MaTIFY10*, *MaTIFY16*, *MaTIFY20*, *MaTIFY25*, *MaTIFY26*, *MaTIFY29*, and *MaTIFY30* exhibited upregulation under cold stress, though the magnitude of induction was relatively modest and variable across the temperature gradient. In the cold-tolerant DJ cultivar, a more robust and sustained transcriptional activation was evident. Genes including *MaTIFY5*, *MaTIFY6*, *MaTIFY7*, *MaTIFY10*, *MaTIFY16*, *MaTIFY17*, *MaTIFY20*, *MaTIFY25*, *MaTIFY26*, *MaTIFY29*, *MaTIFY30*, and *MaTIFY37* displayed pronounced upregulation, particularly at lower temperatures (10 °C and 7 °C), suggesting their involvement in adaptive cold tolerance mechanisms. The downregulated gene set in DJ (*MaTIFY3*, *MaTIFY9*, *MaTIFY12*, *MaTIFY14*, *MaTIFY18*, *MaTIFY19*, *MaTIFY23*, *MaTIFY27*, *MaTIFY32*, *MaTIFY33*, *MaTIFY34*, and *MaTIFY40*) largely overlapped with that in BX; however, the extent of repression differed between cultivars, with DJ generally exhibiting weaker repression for several of these genes. Comparative analysis revealed that *MaTIFY5*, *MaTIFY6*, *MaTIFY7*, *MaTIFY10*, *MaTIFY16*, *MaTIFY20*, *MaTIFY25*, *MaTIFY26*, *MaTIFY29*, and *MaTIFY30* were commonly upregulated in both genotypes under cold stress, yet their expression levels were consistently higher and more sustained in the tolerant DJ cultivar, especially at 7 °C.

### 2.9. Functional Characterization of Drought-Responsive MaTIFY20 Genes

**Figure 8:**
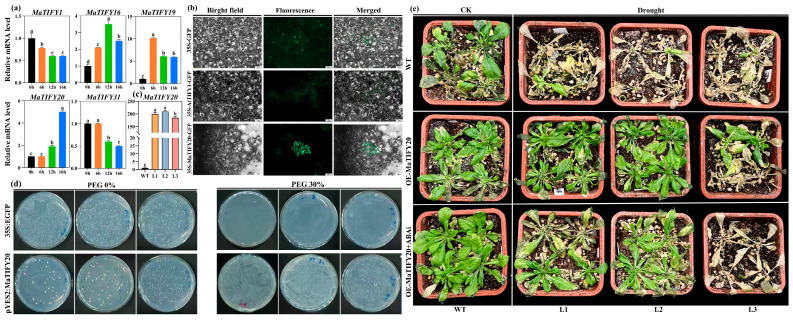
Functional characterization of drought-responsive *MaTIFY* genes. (**a**) Quantitative real-time PCR (qRT-PCR) analysis of *MaTIFY1*, *MaTIFY16*, *MaTIFY19*, *MaTIFY20*, and *MaTIFY31* expression in banana roots under natural drought stress at 0, 6, 12, and 16 h. Data are presented as means ± SD (n = 3). Different letters indicate significant differences (*p* < 0.05). (**b**) Subcellular localization of 35S::GFP (control), 35S::AtTIFY1-GFP, and 35S::MaTIFY20-GFP transiently expressed in tobacco (*Nicotiana benthamiana*) epidermal cells. Scale bar = 20 μm. (**c**) PCR confirmation of a transgenic Arabidopsis plant carrying MaTIFY20 (**d**). Heterologous expression of MaTIFY20 in yeast. Yeast cells transformed with pYES2-MaTIFY20 or empty vector pYES2-EGFP were grown on medium containing 0% or 30% PEG. (**e**) Phenotypic comparison of wild-type (WT) Arabidopsis, *MaTIFY20*-overexpressing (OE-*MaTIFY20*) transgenic lines under normal watering and drought stress conditions, and OE-*MaTIFY20* plants treated with ABA inhibitor (OE-MaTIFY20+ABAI) under control (CK) and drought stress conditions.

To validate the drought-responsive expression of candidate *MaTIFY* genes, quantitative real-time PCR (qRT-PCR) was performed on banana roots subjected to natural drought stress (Figure 8a). The expression of *MaTIFY16*, *MaTIFY19*, and *MaTIFY20* was significantly upregulated under drought conditions, peaking at 12 h or 16 h post-treatment. Notably, *MaTIFY20* exhibited the most dramatic transcriptional induction, with its relative expression increasing approximately 4.5-fold at 16 h compared to the 0 h control. *MaTIFY16* and *MaTIFY19* also showed robust upregulation, reaching peak levels at 12 h and 16 h, respectively. In contrast, *MaTIFY31* displayed relatively lower expression.

Subcellular localization analysis was conducted to determine the cellular compartments in which MaTIFY proteins function. When transiently expressed as C-terminal EGFP fusions in tobacco epidermal cells, both 35S::AtTIFY1-GFP and 35S::MaTIFY20-GFP produced distinct green fluorescent signals exclusively within the nucleus, as evidenced by the merged images showing clear nuclear localization (Figure 8b). The 35S::GFP control displayed diffuse fluorescence throughout the cytoplasm and nucleus. These observations indicate that AtTIFY1 and MaTIFY20 are nuclear-localized proteins, consistent with their predicted roles as transcriptional regulators. PCR confirmation revealed that the transgene was present in MaTIFY20-OE lines (Figure 8c).

To initially assess the functional role of *MaTIFY20* in stress tolerance, a heterologous yeast expression system was employed (Figure 8d). Yeast cells transformed with the pYES2-*MaTIFY20* construct exhibited markedly enhanced growth on medium supplemented with 30% PEG compared to those harboring the pYES2-EGFP control vector, on which control yeast growth was severely inhibited. This result demonstrates that heterologous expression of *MaTIFY20* confers enhanced osmotic stress tolerance in yeast.

Phenotypic analysis of *MaTIFY20*-overexpressing Arabidopsis under drought stress revealed improved tolerance compared to WT plants, as evidenced by reduced leaf wilting and better overall plant vigor (Figure 8e). Interestingly, the ABA inhibitor-treated plants showed compromised drought tolerance even when overexpressing *MaTIFY20*, suggesting that ABA signaling is required for *MaTIFY20*-mediated drought stress responses.

### 2.10. MaTIFY20 Improved Drought Tolerance via Antioxidant Enzyme Modulation

**Figure 9:**
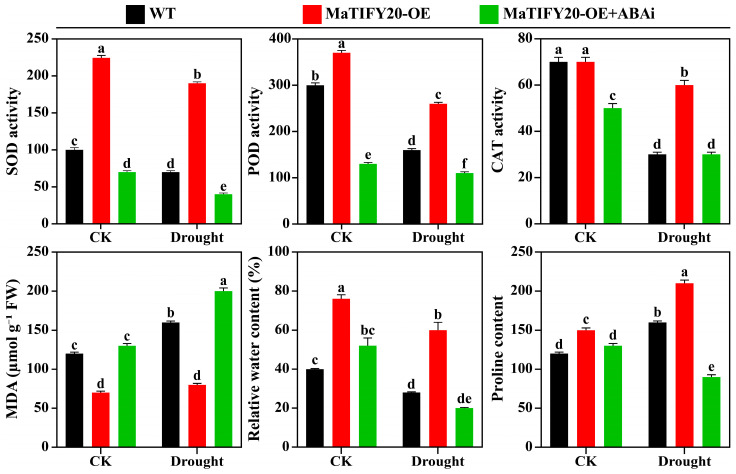
Physiological characterization of MaTIFY20-overexpressing Arabidopsis under drought stress. Antioxidant enzyme activities including SOD (SOD, U g^−1^ FW), POD (SOD, U g^−1^ FW), and (CAT (SOD, U g^−1^ FW); MDA equivalents (nmol g^−1^ FW); RWC (%); and proline content (μg g^−1^ FW) were measured in wild-type (WT), MaTIFY20-overexpressing (MaTIFY20-OE), and MaTIFY20-OE combined with ABA inhibitor (MaTIFY20-OE+ABAI) plants under control (CK) and drought conditions (12 days of water withholding, soil moisture ~30%). Data are presented as means ± SD. Different letters indicate significant differences (*p* < 0.05).

Antioxidant enzymes were measured to further understand the response of MaTIFY20 overexpressing plants to drought stress. Under drought stress, *MaTIFY20*-overexpressing Arabidopsis plants exhibited significantly enhanced antioxidant enzyme activities compared to WT (Figure 9). Superoxide dismutase (SOD), peroxidase (POD), and catalase (CAT) activities were markedly elevated in OE plants under both control and drought conditions, whereas these enhancements were partially attenuated in the MaTIFY20-OE plus ABA inhibitor (MaTIFY20-OE+ABAI) group. Consistent with improved antioxidant capacity, malondialdehyde (MDA) content, a marker of membrane lipid peroxidation, was significantly lower in *MaTIFY20*-OE plants under drought stress relative to WT, but this reduction was compromised in the ABA-inhibited background. Additionally, *MaTIFY20*-OE plants maintained significantly higher relative water content (RWC) and higher proline levels under drought stress than WT; however, these drought-tolerance-associated physiological advantages were diminished when ABA signaling was inhibited.

## 3. Discussion

The TIFY gene family represents a group of plant-specific transcriptional regulators that play pivotal roles in JA signaling, plant development, and responses to both biotic and abiotic stresses [7].

### 3.1. TIFY Genes Are Widely Distributed in the Banana Genome

In this study, we conducted a genome-wide identification and comprehensive characterization of the TIFY gene family in banana, revealing 47 *MaTIFY* members distributed across all 11 chromosomes. The family size of banana TIFY genes (Table 1) is considerably larger than that of *A. thaliana* (18 genes) [18], *Oryza sativa* (20 genes) [20], and *Capsicum annuum* (16 genes) [21]. This expansion suggests that the TIFY family has undergone significant duplication and diversification during banana genome evolution, potentially reflecting the complex regulatory demands of this tropical monocot species.

Phylogenetic analysis partitioned the MaTIFY superfamily into four distinct subfamilies: TIFY, ZIZ/ZML, PPD, and JAZ (Figure 2a), consistent with the established classification in *A. thaliana* [18]. The JAZ subfamily represented the largest clade and was further subdivided into three subgroups (JAZI, JAZII, and JAZIII), mirroring the evolutionary organization observed in other plant species [3]. The high sequence conservation and close clustering of most MaTIFY proteins with their *A. thaliana* counterparts indicate probable functional orthology, suggesting that the core molecular functions of TIFY proteins have been largely preserved during the evolutionary divergence of monocots and dicots [7]. Domain architecture analysis confirmed that all MaTIFY members contained the canonical N-terminal TIFY domain (PF06200) (Figure 2b), while a subset also harbors the CCT_2 domain (PF06203), implying functional diversification through domain shuffling during the course of evolution [20].

The PPI network analysis revealed that key banana TIFY proteins, including *MaTIFY6*, *MaTIFY10*, *MaTIFY3*, and *MaTIFY5*, occupy central hub positions within the predicted interaction network (Figure 5), establishing connections with orthologous partners such as BHLH13 (the putative MYC3 ortholog), GATA25, AFP2, and JAZ7. The topological similarity between the banana and Arabidopsis TIFY networks indicates substantial conservation of interaction partnerships across these two species. The predicted associations with bHLH transcription factors are particularly noteworthy, as MYC2/MYC3-type bHLH proteins are well-established direct interactors of JAZ proteins and serve as master regulators of JA-responsive gene expression [3,7]. The dense interconnections among MaTIFY proteins and their predicted associations with transcription factors and signaling components support the hypothesis that banana TIFY genes operate within conserved jasmonate signaling and transcriptional regulatory cascades.

The *cis*-acting regulatory element analysis of *MaTIFY* promoter regions revealed a complex landscape of hormone-responsive, light-responsive, and stress-responsive elements. The widespread distribution of ABRE and MBS (drought-responsive) elements across *MaTIFY* promoters suggests broad involvement of these genes in ABA-mediated and drought stress signaling pathways (Figure 6). This is consistent with previous reports in wheat and pepper, where TIFY genes were shown to be responsive to multiple abiotic stresses and phytohormone treatments [8,21]. The differential composition and abundance of *cis*-elements among individual *MaTIFY* members indicate that each gene is subject to distinct regulatory inputs, which may underlie their functional specialization in response to diverse environmental and hormonal cues.

### 3.2. MaTIFY Regulates Banana Response to Drought, Cold, and Foc 4 Stress

Expression profiling under drought, *Foc* 4, and cold stress conditions revealed diverse, genotype-specific transcriptional responses among *MaTIFY* genes. Under drought stress, a subset of genes including *MaTIFY3*, *MaTIFY5*, *MaTIFY9*, *MaTIFY16*, *MaTIFY20*, and *MaTIFY25* displayed upregulation, whereas others such as *MaTIFY11*, *MaTIFY14*, *MaTIFY23*, and *MaTIFY27* were significantly downregulated (Figure 7a). This bidirectional transcriptional regulation is consistent with the dual role of JAZ proteins as both positive and negative regulators of stress responses, depending on the specific signaling context and interacting partners [7,22,23]. A recent study in *Osmanthus fragrans (Of)* revealed that TIFY regulates its response to drought and salt stress [24]. The overexpression of *OfJAZ2* reduced plant tolerance to drought and salt stress by inhibiting flavonoid biosynthesis [24]. Meanwhile, the overexpression of *ZmJAZ13* enhanced maize tolerance to drought stress by increasing antioxidant activities and reducing MDA content [18]. In our study, heterologous overexpression of *MaTIFY20* significantly enhanced Arabidopsis tolerance to drought stress (Figure 8e) by increasing antioxidant enzyme activity and proline content (Figure 9). It can be suggested that *MaTIFY20* is a potential drought stress regulator in banana; however, stable transformation in banana would be required to further strengthen our understanding.

The differential expression patterns observed between the susceptible BX and the resistant YK cultivars under *Foc* 4 infection (Figure 7b) highlighted the potential contribution of specific *MaTIFY* genes to disease resistance. The enhanced and sustained induction of *MaTIFY5*, *MaTIFY6*, *MaTIFY10*, *MaTIFY16*, *MaTIFY20*, *MaTIFY26*, *MaTIFY29*, and *MaTIFY30* in the resistant YK cultivar, compared to the susceptible BX cultivar (Figure 7b), suggests that the amplitude and dynamics of MaTIFY-mediated transcriptional reprogramming are critical determinants of resistance to Fusarium wilt [23,25]. This pattern aligns with emerging evidence that the JAZ–MYC2–MYB regulatory hub coordinates defense–growth trade-offs during biotic stress [26]. In *Camellia sinensis*, for instance, the CsMYC2–CsJAZ–CsMYB184 module mediates both development-dependent caffeine accumulation and JA-induced caffeine biosynthesis upon fungal infection, while antagonistic crosstalk between JA and gibberellin signaling via *CsDELLAs–CsJAZ–CsMYB184* and *CsMYC2–CsGA2ox4* interactions optimizes the growth–defense equilibrium [26]. Collectively, these findings suggest that the differential *MaTIFY* expression observed in resistant banana cultivars may reflect a conserved regulatory architecture underlying stress-responsive transcriptional reprogramming across plant species.

Under low-temperature treatment, similar genotype-specific differences were observed between the cold-sensitive BX and the cold-tolerant DJ cultivars (Figure 7c). Genes including *MaTIFY5*, *MaTIFY6*, *MaTIFY7*, *MaTIFY10*, *MaTIFY16*, *MaTIFY20*, *MaTIFY25*, *MaTIFY26*, *MaTIFY29*, and *MaTIFY30* were commonly upregulated in both genotypes, yet their expression levels were consistently higher and more sustained in the tolerant DJ cultivar, particularly at 7 °C. These findings agree with previous studies demonstrating that JAZ proteins can modulate cold stress responses through interaction with ICE1-CBF cold-signaling pathways [7,27,28]. Together, these findings indicate that the enhanced and prolonged MaTIFY induction in DJ reflects a more efficient deployment of a conserved JAZ-dependent regulatory module that integrates hormonal signaling with redox homeostasis to optimize cold stress resilience.

## 4. Materials and Methods

### 4.1. Plant Material and Treatments

The banana plant material used in this study was ‘BX Banana’ (*M. acuminata*, AAA group) along with other cultivars. Plants were grown for 4–6 weeks under controlled greenhouse conditions (28 °C/25 °C day/night, 70% relative humidity, 16 h light/8 h dark photoperiod, 200 μmol m^−2^ s^−1^ light intensity) before stress treatments were applied. For drought stress, banana seedlings were grown in pots containing a mixture of peat and vermiculite (3:1, *v*/*v*) and subjected to progressive soil drying by withholding irrigation. The drought treatment was maintained until day 10, at which point plants exhibited visible wilting, and the soil moisture content decreased to approximately 30% of field capacity. Control plants were maintained under normal watering conditions (soil moisture at 70–80% of field capacity). Leaf tissues were harvested from both drought-stressed and control plants at designated time points for RNA extraction. For *Foc* 4 infection, banana roots were inoculated with *Foc* 4 spore suspension (10^6^ conidia mL^−1^), and root tissues were collected at designated time points post-inoculation for RNA isolation. For cold stress, banana plants were exposed to low-temperature treatments at 16 °C, 10 °C, and 7 °C, and leaf tissues were harvested for RNA extraction. All RNA samples were extracted immediately after the respective stress treatments, verified for integrity (OD_260_/_280_ ratio 1.8–2.0), and stored at −80 °C until use. These previously isolated RNA samples were used for the transcriptomic and qRT-PCR analyses presented in this study.

Wild-type (WT) Arabidopsis thaliana ecotype Columbia-0 (Col-0) was used as the background for transformation and as the control in all experiments. The MaTIFY20 overexpression construct was generated by cloning the full-length coding sequence (CDS) of MaTIFY20 into the binary vector pCAMBIA1302 under the control of the CaMV35S promoter, with GFP as a C-terminal fusion tag for confirmation of expression. The construct was introduced into *Agrobacterium tumefaciens* strain GV3101 and transformed into WT Col-0 plants via the floral dip method. For drought stress assays, T3 homozygous transgenic seeds were sown in pots containing a peat: vermiculite mixture (3:1, *v*/*v*) and grown under controlled conditions (22 °C/18 °C, day/night; 60% relative humidity; 16 h light/8 h dark photoperiod). Drought stress was imposed by withholding irrigation for 12 days until the soil moisture content decreased to approximately 30% of field capacity, at which point plants exhibited visible wilting. Control plants were maintained at field capacity by regular watering throughout the experiment.

To investigate whether ABA signaling is required for MaTIFY20-mediated drought stress responses, the ABA biosynthesis inhibitor fluridone (Sigma-Aldrich, St. Louis, MO, USA; CAS No. 59756-60-4) was used. Fluridone was dissolved in dimethyl sulfoxide (DMSO) to prepare a 10 mM stock solution, which was diluted to a final working concentration of 10 μM in ½ MS liquid medium. For soil-grown plants, fluridone was applied as a soil drench at 10 μM (10 mL per pot) every 3 days, beginning 7 days before the initiation of drought stress and continuing throughout the drought treatment period. The control group (WT and OE-MaTIFY20 without inhibitor) received an equivalent volume of 0.1% (*v*/*v*) DMSO in ½ MS medium as a mock treatment. The OE-MaTIFY20+ABAI group refers to MaTIFY20-overexpressing plants treated with 10 μM fluridone.

### 4.2. Genomic Data Sources

Genome sequences and annotation files for *M. acuminata* DH-Pahang V4 and Baxijiao V1 were downloaded from the Banana Genome Hub (https://banana-genome-hub.southgreen.fr/, accessed on 10 April 2026). Protein sequences of TIFY family members from *Arabidopsis thaliana* were retrieved from TAIR (https://www.arabidopsis.org/, accessed on 10 April 2026).

### 4.3. Identification of TIFY Family Members and Physicochemical Analysis

The *Arabidopsis* TIFY proteins were used as query sequences, using the BLAST 2.17.0 (E-value < 1 × 10^−5^) algorithm in TBtools-II v2.114 analysis software to identify the TIFY in the banana genome [29]. Duplicate entries were removed, unique hits were retained, and results were summarized. The conserved domain of TIFY proteins was searched using the Pfam website (http://pfam.xfam.org/, accessed on 11 April 2026). Candidate proteins were further screened using NCBI’s Conserved Domain Database (CDD, https://www.ncbi.nlm.nih.gov/cdd, accessed on 12 April 2026) [30]. Physicochemical properties (number of amino acids, molecular weight, theoretical isoelectric point, instability index, aliphatic index, grand average of hydropathicity) of the identified TIFY proteins were predicted using the ProtParam tool on the ExPASy server (https://web.expasy.org/protparam/, accessed on 13 April 2026) [31]. Subcellular localization was predicted using Plant-mPLoc (available within the Cell-PLoc 2.0 suite: http://www.csbio.sjtu.edu.cn/bioinf/plant-multi/, accessed on 23 April 2026) [32]. Secondary structure was predicted using SOPMA (https://npsa-prabi.ibcp.fr/cgi-bin/npsa_automat.pl?page=/NPSA/npsa_sopma.html, accessed on 25 April 2026) [33].

### 4.4. Conserved Motif Analysis

Conserved protein motifs were identified using the MEME suite (https://meme-suite.org/meme/tools/meme, accessed on 5 May 2026) with the following parameters, including maximum number of motifs, motif site, and motif width range [34]. The resulting MEME XML output file was imported into TBtools-II v2.114 for visualization alongside the gene structure.

### 4.5. Phylogenetic Analysis and Protein Sequence Alignment

Full-length protein sequences of TIFYs from banana and Arabidopsis were aligned using the Muscle algorithm implemented in MEGA7 [35]. Phylogenetic trees were constructed using the Neighbor-Joining (NJ) method with 1000 bootstrap replicates. The Poisson model was used, and pairwise deletion was applied to handle missing data. The resulting trees were visualized with the iTOL online platform (https://itol.embl.de/, accessed on 11 May 2026). Amino acid sequence homology between banana and Arabidopsis TIFYs was analyzed using the MegAlign module within the DNASTAR Lasergene 11 software suite [36]. The tertiary structure of the representative MaTIFY protein was predicted using SWISS-MODEL (https://swissmodel.expasy.org/, accessed on 13 May 2026). The template with the highest GMQE score was selected, and the corresponding PDB file was downloaded [37].

### 4.6. Promoter Cis-Acting Element Analysis

Promoter sequences (1500 bp upstream of the ATG) of the *MaTIFY* genes were extracted from the banana genome using TBtools-II v2.114. All sequences were subjected to *cis*-acting element prediction through the PlantCARE online analysis website (https://bioinformatics.psb.ugent.be/webtools/plantcare/html/, accessed on 19 May 2026) [38]. The *cis*-acting elements were sorted using the downloaded prediction file, with blank entries removed. Elements involved in environmental stress responses and hormone signaling were retained. The sorted files are visualized by TBtools-II v2.114.

### 4.7. Prediction of the Interaction Network

psRNATarget (https://www.zhaolab.org/psRNATarget/, accessed on 16 May 2026) and PlantTFDB (http://planttfdb.gao-lab.org/, accessed on 17 May 2026) were used to predict miRNAs and the transcription factors that regulate *MaTIFY*, respectively [39,40]. The miRNA prediction expected value was set to 5, the transcription factor binding site prediction parameter was set to *p*-value ≤ 1 × 10^−6^, and the remaining parameters were set to default values. The results were visualized using Cytoscape 3.10.3 software [41]. The STRING data in the transcriptome were used to screen proteins with known biological functions and potential interactions with MaTIFY20. The screening results were imported into Cytoscape 3.10.3 software for network visualization [42].

### 4.8. Subcellular Localization

First-strand cDNA was synthesized from RNA of four treated root samples using RevertAid Master Mix (Thermo Electron Corp, Waltham, MA, USA). The full-length MaTIFY20 CDS was amplified using 2× SanTaq PCR Master Mix with primers listed in Table S1 (95 °C, 3 min; 34 cycles of 95 °C, 30 s; 63 °C, 30 s; 72 °C, 2 min; final extension 72 °C, 10 min). The product was gel-purified, ligated into pMD18-T (Takara, Dalian, China), and transformed into DH5α. Positive clones were verified by sequencing.

The insert and pRI101-AN were digested with Sal I/Kpn I, ligated, and transformed into DH5α. The recombinant pRI101-MaTIFY20-EGFP was verified and introduced into *Agrobacterium tumefaciens* GV3101. Transformants were resuspended in infiltration medium (MS + 100 mM MgCl_2_ + 100 μM acetosyringone + 5% sucrose) to OD_600_ = 1.0 and infiltrated into *N. benthamiana*. After 2 days in darkness at 28 °C, cells were stained with DAPI and examined under a confocal microscope (Olympus FV1200, Tokyo, Japan) to determine MaTIFY20 subcellular localization.

### 4.9. RNA Extraction and qRT-PCR Analysis

We extracted total RNA from banana and Arabidopsis leaf tissues with the RNAprep Pure Plant Kit (Tiangen, Beijing, China) and performed reverse transcription using HiScript II Q RT SuperMix (Vazyme, Nanjing, China). qPCR was then carried out with the SYBR Green PCR Master Mix kit (TransGen, Beijing, China). Gene expression was quantified by the 2^−ΔΔCT method following [14], and the *MaActin1* gene was selected as the calibration gene. Primer sequences are provided in Supplementary Table S1.

### 4.10. Determination of Antioxidant Enzyme Activities and Physiological Parameters

Antioxidant enzyme assays. Fresh leaf tissues (0.5 g) from wild-type (WT), MaTIFY20-overexpressing (OE), and OE+ABAI Arabidopsis plants were homogenized in 5 mL of ice-cold extraction buffer containing 50 mM potassium phosphate buffer (pH 7.8), 0.1 mM EDTA, 1% (*w*/*v*) polyvinylpyrrolidone (PVP), and 0.1% (*v*/*v*) Triton X-100. The homogenate was centrifuged at 12,000× *g* for 15 min at 4 °C, and the supernatant was used immediately for enzyme activity assays.

SOD activity was assayed by monitoring the photochemical reduction of nitroblue tetrazolium (NBT) at 560 nm. One unit of SOD activity was defined as the amount of enzyme required to inhibit 50% of the NBT photoreduction rate in a 3 mL reaction mixture containing 50 mM potassium phosphate buffer (pH 7.8), 13 mM methionine, 75 μM NBT, 2 μM riboflavin, 0.1 mM EDTA, and 50 μL of enzyme extract. The reaction was initiated by exposing the tubes to a 30 W fluorescent lamp for 15 min, and absorbance was measured at 560 nm using a spectrophotometer (UV-2600, Shimadzu, Kyoto, Japan).

POD activity was determined using the guaiacol oxidation method with minor modifications. The 3 mL reaction mixture contained 50 mM potassium phosphate buffer (pH 7.0), 20 mM guaiacol, 10 mM H_2_O_2_, and 50 μL of enzyme extract. The increase in absorbance due to the formation of tetraguaiacol was recorded at 470 nm for 3 min at 25 °C. POD activity was calculated using the extinction coefficient (ε = 26.6 mM^−1^ cm^−1^) and expressed as μmol min^−1^ g^−1^ fresh weight (FW).

CAT activity was measured by monitoring the decomposition of H_2_O_2_ at 240 nm (ε = 39.4 M^−1^ cm^−1^). The reaction mixture (3 mL) contained 50 mM potassium phosphate buffer (pH 7.0), 10 mM H_2_O_2_, and 50 μL of enzyme extract. The decrease in absorbance was recorded at 240 nm for 3 min at 25 °C. CAT activity was expressed as μmol H_2_O_2_ decomposed min^−1^ g^−1^ FW.

MDA content was determined as a marker of lipid peroxidation using the thiobarbituric acid (TBA) reaction method with slight modifications. Leaf tissue (0.5 g) was homogenized in 5 mL of 10% (*w*/*v*) trichloroacetic acid (TCA) and centrifuged at 12,000× *g* for 15 min at 4 °C. A 2 mL aliquot of the supernatant was mixed with 2 mL of 0.6% (*w*/*v*) TBA in 10% TCA, heated at 95 °C for 30 min, and then rapidly cooled in an ice bath. After centrifugation at 10,000× *g* for 10 min, the absorbance of the supernatant was measured at 532 nm and corrected for non-specific turbidity by subtracting the absorbance at 600 nm. The MDA concentration was calculated using an extinction coefficient of 155 mM^−1^ cm^−1^ and expressed as nmol g^−1^ FW.

Free proline content was estimated using the acid ninhydrin method. Leaf tissue (0.5 g) was homogenized in 5 mL of 3% (*w*/*v*) sulfosalicylic acid and centrifuged at 10,000× *g* for 10 min. The reaction mixture contained 2 mL of supernatant, 2 mL of acid ninhydrin reagent (1.25 g ninhydrin in 30 mL glacial acetic acid and 20 mL 6 M phosphoric acid), and 2 mL of glacial acetic acid. The mixture was incubated at 95 °C for 30 min, cooled on ice, and extracted with 4 mL of toluene. The absorbance of the pink-colored toluene phase was measured at 520 nm. Proline content was calculated using a standard curve and expressed as μg g^−1^ FW.

RWC was determined using the frequently used method. Fresh leaf discs were weighed immediately to obtain fresh weight (FW), then floated on distilled water for 24 h at 25 °C in the dark to obtain turgid weight (TW). Subsequently, the samples were oven-dried at 105 °C for 24 h to determine dry weight (DW). RWC was calculated as:

$$\mathrm{RWC}(\%)\;=\;\frac{FW-DW}{TW-DW}\times 100$$

### 4.11. Yeast Expression Assay

The cDNA of *MaTIFY20* was subcloned into the yeast expression vector pYES2. The resulting plasmid, pYES2-MaTIFY20, was used to transform strain W303a by the lithium acetate method, and the transformants were selected on SD medium (-uracil). Yeast cells carrying the MaTIFY20 protein were incubated for 3 days at 30 °C, harvested, and diluted to an OD600 value of 0.1 and 0.01 with distilled water. Five microliters of yeast cultures were transferred onto SD-Gal (2% galactose) solid medium supplemented with 0% or 30% (*w*/*v*) PEG6000 to induce osmotic stress. The plates were incubated at 30 °C for 2–3 days before being photographed.

### 4.12. Statistical Analysis

All quantitative data are presented as means ± standard deviation (SD), based on a minimum of three biological replicates. Statistical analyses were conducted using Origin Pro 2024 and Microsoft Excel 365. Data normality was evaluated using the Shapiro–Wilk test. For comparisons between two groups, Student’s t-test was used, while one-way analysis of variance (ANOVA) followed by Tukey’s post hoc test was used for multiple-group comparisons. A *p*-value less than 0.05 was considered statistically significant.

## 5. Conclusions

In this study, we identified 47 *MaTIFY* genes in banana (*M. acuminata*) and systematically characterized their phylogenetic relationships, conserved motifs, *cis*-regulatory elements, and expression profiles under drought, *Foc* 4, and cold stresses. The *MaTIFY* family expanded through tandem and segmental duplications, with members classified into four subfamilies (TIFY, ZIZ/ZML, PPD, and JAZ). Stress-responsive expression analysis revealed that *MaTIFY5*, *MaTIFY20*, *MaTIFY26*, *MaTIFY29*, and *MaTIFY30* were differentially induced in resistant cultivars, suggesting their involvement in stress adaptation. Functional validation of *MaTIFY20* confirmed its drought-inducible expression, nuclear localization, and positive role in osmotic stress tolerance. These findings, together with the predicted involvement of *MaTIFY* genes in JA-mediated signaling pathways, provide candidate genes and a theoretical foundation for molecular breeding of banana cultivars with enhanced resistance to abiotic and biotic stresses. Further functional studies are needed to validate the specific regulatory roles of individual *MaTIFY* members.

## Figures and Tables

**Table 1:** Physicochemical properties of the *MaTIFY* gene family in Banana.

Locus ID	Name	Chr.	CDS	AA	MW (kDa)	PI	SL
Macma4_01_g15860	*MaTIFY1*	1	1062	353	38,138.39	9.48	Nucleus
Macma4_02_g11690	*MaTIFY2*	1	1311	436	46,593.05	6.43	Nucleus
Macma4_02_g12310	*MaTIFY3*	2	1065	355	38,455.56	6.76	Nucleus
Macma4_02_g16390	*MaTIFY4*	2	414	137	15,610.71	7.68	Nucleus
Macma4_02_g25340	*MaTIFY5*	2	672	223	24,285.68	9.65	Nucleus
Macma4_03_g01960	*MaTIFY6*	3	630	209	22,948.2	9.71	Nucleus
Macma4_03_g06070	*MaTIFY7*	3	756	251	26,977.33	8.93	Nucleus
Macma4_03_g10240	*MaTIFY8*	3	693	230	25,255.6	7.03	Nucleus
Macma4_03_g16810	*MaTIFY9*	3	1197	398	42,048.99	8.24	Nucleus
Macma4_03_g25970	*MaTIFY10*	3	630	209	23,213.58	10.32	Nucleus
Macma4_04_g09960	*MaTIFY11*	4	741	246	25,561.92	8.26	Nucleus
Macma4_04_g40350	*MaTIFY12*	4	456	151	17,138.53	9.51	Nucleus
Macma4_05_g05150	*MaTIFY13*	5	726	241	26,309.73	7.67	Nucleus
Macma4_05_g05960	*MaTIFY14*	5	1248	415	43,596.18	9.66	Nucleus
Macma4_05_g25430	*MaTIFY15*	5	465	164	17,852.62	10.7	Nucleus
Macma4_05_g25520	*MaTIFY16*	5	753	250	27,083.43	9.15	Nucleus
Macma4_05_g28320	*MaTIFY17*	5	477	158	16,662.92	5.91	Nucleus
Macma4_06_g03500	*MaTIFY18*	6	1092	363	38,319.56	6.7	Nucleus
Macma4_06_g12690	*MaTIFY19*	6	1164	387	43,127.91	9.16	Nucleus
Macma4_06_g22320	*MaTIFY20*	6	483	160	17,647.43	9.93	Nucleus
Macma4_06_g34370	*MaTIFY21*	6	642	213	23,265.3	6.65	Nucleus
Macma4_06_g34540	*MaTIFY22*	6	450	149	17,123.76	9.78	Nucleus
Macma4_07_g01250	*MaTIFY23*	7	402	133	14,733	9.42	Nucleus
Macma4_07_g01260	*MaTIFY24*	7	477	158	17,778.99	8.51	Nucleus
Macma4_07_g12510	*MaTIFY25*	7	1233	410	43,541.82	9.47	Nucleus
Macma4_07_g15770	*MaTIFY26*	7	534	177	18,819.65	9.93	Nucleus
Macma4_07_g22660	*MaTIFY27*	7	729	242	26,079.27	8.39	Nucleus
Macma4_08_g00550	*MaTIFY28*	8	522	173	18,821.67	6.3	Nucleus
Macma4_08_g04330	*MaTIFY29*	8	1158	385	41,235.87	9.09	Nucleus
Macma4_08_g06910	*MaTIFY30*	8	456	151	16,244.31	6.73	Nucleus
Macma4_08_g19160	*MaTIFY31*	8	762	253	26,626.03	9.35	Nucleus
Macma4_08_g25200	*MaTIFY32*	8	558	185	19,625.35	6.74	Nucleus
Macma4_08_g25320	*MaTIFY33*	8	387	128	14,862.14	10.4	Nucleus
Macma4_08_g26190	*MaTIFY34*	8	687	228	23,181.2	9.62	Nucleus
Macma4_08_g28410	*MaTIFY35*	8	1083	360	39,111.1	7.57	Nucleus
Macma4_09_g01110	*MaTIFY36*	9	1059	352	39,243.73	9.36	Nucleus
Macma4_09_g10730	*MaTIFY37*	9	384	127	14,512.76	9.62	Nucleus
Macma4_09_g14380	*MaTIFY38*	9	318	105	10,823.49	6.7	Nucleus
Macma4_09_g14550	*MaTIFY39*	9	798	265	28,932.58	8.61	Nucleus
Macma4_09_g15840	*MaTIFY40*	9	1233	410	44,153.33	9	Nucleus
Macma4_09_g21220	*MaTIFY41*	9	417	138	15,260.68	9.13	Nucleus
Macma4_10_g06730	*MaTIFY42*	10	744	247	26,465.73	9.38	Nucleus
Macma4_10_g06810	*MaTIFY43*	10	534	177	19,015.75	9.3	Nucleus
Macma4_10_g21960	*MaTIFY44*	10	1059	352	37,826.28	8.95	Nucleus
Macma4_10_g22390	*MaTIFY45*	10	576	191	21,053.11	10.29	Nucleus
Macma4_10_g35050	*MaTIFY46*	10	603	200	22,537.64	9.83	Nucleus
Macma4_11_g09370	*MaTIFY47*	11	780	259	27,808.45	9.46	Nucleus

Coding Sequence: CDS, Chromosome: Chr, Molecular Weight: MW, Isoelectric point: PI, and Sub-Cellular Location: SL.

## Data Availability

The original contributions presented in this study are included in the article. Further inquiries can be directed to the corresponding authors.

## Supplementary Materials

The following supporting information can be downloaded at: https://www.mdpi.com/article/10.3390/ijms27157044/s1.
