# Supplementary material for: Evolutionary Analysis and Expression Profiling of the TIFY Gene Family in Banana Under Multiple Stresses and Functional Characterization of MaTIFY20 in Drought Tolerance
Source: Int J Mol Sci. 2026 Aug 6;27(15):7044. doi: 10.3390/ijms27157044 (PMC13466436; doi:10.3390/ijms27157044)
Supplement: Supplementary file 1 [file ijms-27-07044-s001.zip › ijms-4455334-Supplementary.pdf]

Table S1. List of the primers used in the study.

Gene	Primer sequence 5'-3'
	**qRT-PCR Primers**
MaActin-F	CTTAGCACTTTCCAGCAGATG
MaActin-R	ACACCAAAAAACTACCCCGAC
MaTIFY1-F	TTCTGGCTCAACTTAGGCCG
MaTIFY1-R	ACATGTGCAGTTTGCTGGTC
MaTIFY16-F	ACTTGAAGGAGAAGGGCAGC
MaTIFY16-R	CAGTGGACACATCCACTCCC
MaTIFY19-F	AGGCTTGCACAGGCTACATT
MaTIFY19-R	GGTTCGAATCGGACTGCTCA
MaTIFY20-F	AGATCAGCGGGAAAGGGTTG
MaTIFY20-R	CCTCTCTTTGCGCTTCTGGA
	**Overexpression primer**
MaTIFY20-F	CACCCATGGATGTCGAGAGATGTCGTGGAATTCGATTTCTTTGCG
MaTIFY20-R	GAGAGATCTAACACGTATTCTTAGCCGAACCCGTCGCC
	**Yeast overexpression**
MaTIFY20-F	CGCGAATTCATGTCGAGAGATGTCGTGGAATTCGATTTCTTTGCG
MaTIFY20-R	CCGCTCGAGAACACGTATTCTTAGCCGAACCCGTC
	**Subcellular localization**
MaTIFY20-F	CACCCATGGATGTCGAGAGATGTCGTGGAATTCGATTTCTTTGCG
MaTIFY20-R	GAGAGATCTAACACGTATTCTTAGCCGAACCCGTC

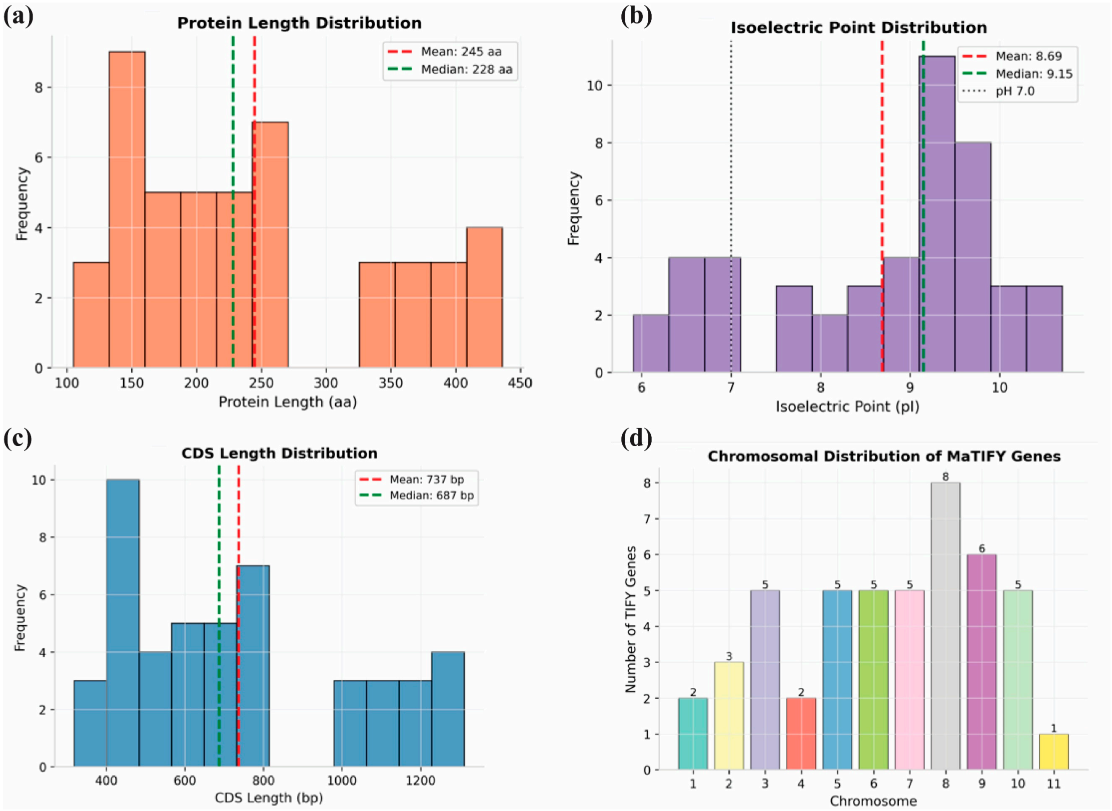

Figure S1. Chemical and physical properties of MaTIFY proteins.

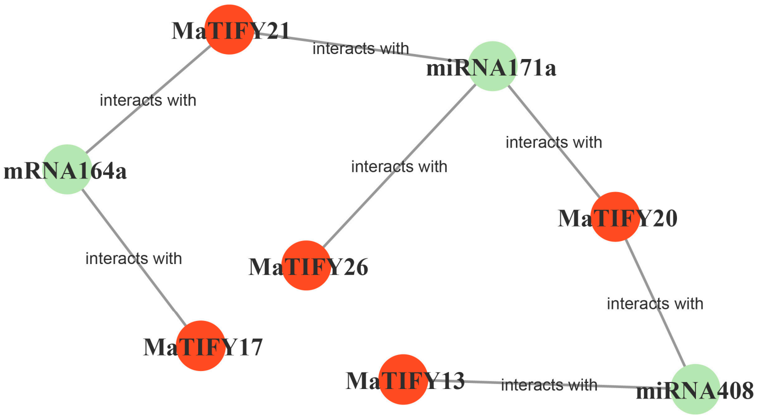

Figure S2. miRNA-MaTIFY network.
